# Chemical Characterization of Honeysuckle Polyphenols and Their Alleviating Function on Ultraviolet B-Damaged HaCaT Cells by Modulating the Nrf2/NF-κB Signaling Pathways

**DOI:** 10.3390/antiox13030294

**Published:** 2024-02-28

**Authors:** Shuo-Lei Zheng, Yu-Mei Wang, Chang-Feng Chi, Bin Wang

**Affiliations:** 1 Zhejiang Provincial Engineering Technology Research Center of Marine Biomedical Products, School of Food and Pharmacy, Zhejiang Ocean University, Zhoushan 316022, China; 2 National and Provincial Joint Engineering Research Centre for Marine Germplasm Resources Exploration and Utilization, School of Marine Science and Technology, Zhejiang Ocean University, Zhoushan 316022, China

**Keywords:** honeysuckle polyphenols (HPs), LC-MS/MS, ultraviolet B radiation, oxidative stress, Nrf2/NF-κB (P65) pathways, molecular docking

## Abstract

Scientific evidence attests that the epidermis receives excessive ultraviolet B (UVB) radiation, triggering the generation of substantial quantities of reactive oxygen species (ROS), which disrupted the delicate equilibrium of oxidation–reduction, leading to oxidative stress and inflammation. The historical use of honeysuckle polyphenols (HPs) has garnered our attention due to their efficacy in inhibiting oxidative damage. In this study, HPs were prepared from honeysuckle flowers employing an ultrasonic-assisted extraction method and quantitatively analyzed by a LC–MS/MS, and the mechanisms underlying HPs’ antioxidative and anti-inflammatory effects on a UVB-irradiated HaCaT cell model were systematically investigated. The results showed that HPs had a significant cellular repair effect on UVB-irradiated HaCaT cells (*p* < 0.001). The mechanism of action indicated that HPs could allow Nrf2 to enter the nucleus by regulating the dissociation of Nrf2 from Keap1, which further increases the activity of downstream proteases (SOD and CAT), increases ROS scavenging, and reduces the intracellular malondialdehyde (MDA) level. In addition, HPs could down-regulate Toll-like receptor 4 (TLR4) and inhibit NF-κB (P65) dissociating from IκBα, resulting in a decrease in NF-κB (P65) entry into the nucleus and a decrease in inflammatory factors (TNF-α, IL-6, and IL-1β). In addition, four key compounds in HPs, including chlorogenic acid, quercetin, isorhamnetin, and luteolin, were selected to verify the mechanism of HPs repairing UVB damage using molecular docking techniques. The experiment suggested that four key active compounds could effectively occupy the Kelch homologue (Kelch) structural domain of Keap1, competitively bind with Nrf2, and facilitate the promotion of Nrf2 binding, ultimately enhancing the translocation of Nrf2 into the nucleus. In addition, four key active compounds could effectively interact with NF-κB (P65) through hydrogen bonding, van der Waals forces, and electrostatic forces to inhibit its entry into the nucleus. In summary, HPs can effectively repair the damage of HaCaT cells by UVB radiation and can be used to develop health and cosmetic products for the treatment of UV radiation-induced diseases.

## 1. Introduction

Ultraviolet (UV) radiation is a major external factor in sunburn, erythema, photodamage, and skin cancer [1,2]. UV radiation is categorized into three types based on wavelength [3]. UVC (315–400 nm) radiation is effectively absorbed by the O_3_ layer and does not reach the Earth’s surface. In comparison, UVB (280–315 nm) radiation, with higher energy than UVA (100–280 nm), stands as the primary culprit behind skin photodamage [4].

Excessive UVB radiation directly damages DNA in the skin and generates high levels of reactive oxygen species (ROS), leading to the activation of antioxidant and inflammatory signaling pathways [5]. ROS assumes a pivotal role in fostering oxidative stress. The skin boasts a robust antioxidative defense system, and the Keap1/Nrf2 signaling pathway emerges as the pivotal component activated in responding to oxidative stress [6]. The uncoupling of Keap1/Nrf2 allows the Nrf2 transcription factor to translocate into the nucleus and activate downstream antioxidant enzymes [7]. Superoxide dismutase (SOD), catalase (CAT), and other enzymes play a critical role in eliminating excess ROS and cellular lipid peroxides, such as malondialdehyde (MDA) [8]. This process contributes to maintaining the balance of oxidative and antioxidant properties within the body, thereby safeguarding skin cells from the potential damage arising from redox imbalance [9]. Moreover, oxidative stress possesses the capacity to instigate inflammatory responses, in which the NF-κB signaling pathway plays a prominent role [10]. Under the influence of oxidative stress, the activation of the membrane receptor protein TLR4 is elicited [11], and this activation triggers the dissociation of NF-κB (P65) from IκBα, resulting in an amplified translocation of NF-κB (P65) into the nucleus [12,13], subsequently culminating in the production of pertinent inflammatory factors, such as TNF-α, IL-6, and IL-1β [14,15].

Honeysuckle is also known as Japanese honeysuckle, and its buds and flowers are widely used as a traditional edible herb in China, Japan, and Korea [16,17]. The buds, leaves, and stems of Honeysuckle show considerable consistency in appearance and chemical composition, including higher levels of hydroxycinnamic acids and flavonoids, which are closely associated with the plant’s anti-inflammatory and antioxidant activities. In pharmacological studies, the buds, leaves, and stems of Honeysuckle showed significant anti-inflammatory activity. These plant parts have been shown to be effective modulators of inflammatory responses through the croton oil-induced mouse ear oedema and carob gum-induced paw oedema tests, providing scientific support for their use as anti-inflammatory agents in traditional herbal medicine. In addition, in experiments with LPS-stimulated RAW 264.7 macrophages, the buds, leaves, and stems exhibited a protective effect on the cells [16]. This suggests that these parts of Honeysuckle may have immunomodulatory and cytoprotective potential, providing a new understanding of their positive role in the treatment of inflammatory diseases.

Honeysuckle polyphenols (HPs) show notable anti-inflammatory, antiviral, anti-tumor, and antioxidant effects [18]. Jeong et al. reported [19] that HPs were verified to possess anti-inflammatory and antioxidant effects through the MAPK/NF-κB pathways. These properties suggest their potential utility in serving as drugs for treating dermatitis. Su et al. reported [20] that the anti-inflammatory attributes of HPs were substantiated through investigations on multiple cellular and animal models of inflammation. Consequently, these significant findings highlight the valuable applications of HPs in antioxidant and anti-inflammatory contexts, underscoring their potential therapeutic relevance in the scientific literature.

Given its capability to precisely predict the conformation of protein–ligand binding sites, molecular docking is frequently employed in the realm of structure-based drug design and the anticipation of functional sites residing on the surface of protein molecules [21]. Nevertheless, the scores presented during docking do not inherently represent the exact binding affinity. Hence, to validate the outcomes of molecular docking, the crucial step of visually confirming the structural binding becomes imperative [22].

In this investigation, the primary constituents within HPs were characterized through LC–MS/MS analysis for subsequent in-depth exploration. In addition, UVB radiation was employed to establish a UVB-damaged skin keratinocytes (HaCaT cells) model for illustrating the mechanism of HPs ameliorating the damage of UVB radiation. This study not only highlights the potential of HPs in improving UV damage but also lays a theoretical foundation for developing HPs as a therapeutic agent for UV damage.

## 2. Materials and Methods

### 2.1. Materials and Chemical Reagents

Dried honeysuckle buds and flowers were purchased from Maonan District, Guangdong Province, China. Methanol, ascorbic acid (VC), bicinchoninic acid (BCA), and 1,1-diphenyl-2-picrylhydrazyl (DPPH) were purchased from Sigma-Aldrich Trading Co., Ltd. (Shanghai, China). The 0.25% EDTA-trypsin and high-efficiency RIPA tissue/cell rapid lysate (with PMSF: protease inhibitor) were purchased from Beijing Solepol Technology Co., Ltd. (Beijing, China). Elisa kits (TNF-α, IL-6, and IL-1β) were purchased from Shanghai Enzyme Link Biotechnology Co., Ltd. (Shanghai, China). HaCaT cells and the culture medium were procured from Wuhan Punosai Life Science and Technology Co., Ltd. (Wuhan, China). The antibodies (Nrf2, Keap1, IκBα, Phospho-IκBα, TLR4, NF-κB(P65) and Phospho-NF-κB(P65)) were purchased from Wuhan Sanying Bio-technology Co., Ltd. (Wuhan, China).

### 2.2. Preparation of Honeysuckle Polyphenols (HPs)

The procedure for extracting HPs [23,24] with ultrasound assistance proceeded as follows: initially, dried honeysuckle buds and flowers underwent pulverization using a DFT-250 portable high-speed universal pulverizer (manufactured by Linda Machinery Co., Ltd., Wenling, Wenzhou, China). Subsequently, honeysuckle bud and flower powder was added to 70% methanol and maintained a material–liquid ratio of 1:20. The resultant mixture was then placed in an ultrasonic apparatus operating at a power of 200 W and a frequency of 35 KHz, and processed at a temperature of 50 °C for 50 min. Upon completion of the ultrasonic treatment, the extracted mixture was filtered to prepare the filtrate, and the methanol solution was removed through rotary evaporation at 50 °C.

Crude HPs were further purified using AB-8 macroporous resin, which consisted of the following steps [25]. Firstly, crude HP solution (6.0 mg/mL) was added into an AB-8 resin column (100 mm × 300 mm) at a flow rate of 3 BV/h, with a maximum operating volume of 160 mL, and an adsorption time of 2 h. Then, the sample was eluted with distilled water until the solution was colorless. Next, the polyphenols were eluted with 95% ethanol at a flow rate of 5 BV/h and an eluent dosage of 5 BV. Subsequently, organic reagents were removed using rotary evaporation. Finally, purified HPs were obtained by vacuum freeze-drying technique.

### 2.3. LC–MS/MS Analysis

The LC–MS/MS analysis of HPs was entrusted to Shanghai Meiji Biomedical Technology Co., Ltd. (Shanghai, China) The Instrument platform for this LC–MS/MS analysis was a Thermo Fisher U-HPLC-Q Exactive HF-X system. The U-HPLC conditions were as follows: Waters ACQUITY UPLC HSS T3 column (100 mm × 2.1 mm, 1.8 µm) (Milford, CT, USA); column temperature was 40 °C; mobile phase A was 95% water + 5% acetonitrile (containing 0.1% formic acid), and mobile phase B was 47.5% acetonitrile + 47.5% isopropanol + 5% water (containing 0.1% formic acid) with isocratic elution (A:B solvent ratio 2:1); injection volume was 3.0 μL. Mass spectrum parameters: scan type (*m*/*z*) 70–1050; sheath gas flow rate (arb) 50; aux gas flow rate (arb) 13; heater temp (°C) 425; capillary temp (°C) 3; spray voltage (+) (V) 3; spray voltage (−)(V) −35; S-lens RF level 50; normalized collision energy (eV) 20, 40, 60; resolution (full MS) 6000; resolution (MS2) 7500.

### 2.4. DPPH Radical Scavenging Activity

HPs were formulated into different concentrations (0, 0.02, 0.04, 0.06, 0.08, 0.10, and 0.20 mg/mL). The experimental groups included A_1_ (HPs + DPPH (0.2 mM) mixed in a 1:1 ratio), A_2_ (HPs + ethanol solution mixed in a 1:1 ratio), and A_0_ (control group with distilled water and DPPH (0.2 mM) mixed in a 1:1 ratio). Additionally, an A_2_ blank group (HPs + ethanol solution mixed in a 1:1 ratio) and an A_0_ control group (distilled water and DPPH (0.2 mM) mixed in a 1:1 ratio) were established. The mixtures were incubated at room temperature for 30 min in the absence of light [26,27], and then the absorbance was measured at 520 nm by a microplate reader (Multiskan FC zymograph).

DPPH radical scavenging rate (%) = (1 − (A_1_ − A_2_)/A_0_) × 100

### 2.5. Cytoprotection of HPs on UVB-Irradiated Cell Model

#### 2.5.1. Cell Culture and Establishment of UVB-Irradiated Cell Model

UVB-irradiated cell model was established according to the previous method [28]. HaCaT cells were cultured at 37 °C in a humidified incubator with 5% CO_2_ supplementation, utilizing HaCaT-specific medium. Cultured at a density of 1 × 10^4^ cells/well, HaCaT cells were seeded in 96-well plates with 100 µL of medium/well. After 24 h, the medium was aspirated, and HaCaT cells were thrice washed with PBS buffer. Subsequently, a thin layer of PBS was applied to cover the HaCaT cells, with non-irradiated wells shielded using tin foil. UVB irradiation, sourced from a 313 nm light source, was administered at doses of 0, 2.2, 3.0, 4.0, and 5.0 mJ/cm^2^, respectively.

Radiation dose (mJ/cm^2^) = Radiation intensity (mw/cm^2^) × Time (s)

After UVB radiation, HaCaT cells were washed three times with PBS and cultured in new medium for 24 h. The cells were then incubated in a humidified incubator at 37 °C with 5% CO_2_. After that, the wells were washed with PBS, CCK8 was added, and they were cultured in a humidified incubator with 5% CO_2_ at 37 °C for 1 h. The cells were then incubated in a humidified incubator with 5% CO_2_ for 1 h and the absorbance at 450 nm was determined.

Cell viability (%) = (OD_sample_/OD_control_) × 100.

In addition, the UVB dose inducing HaCaT cell viability of approximately 50% will be selected for cell modeling.

#### 2.5.2. Effect of HPs on the Viability of UVB-Irradiated Cells

After 24 h of cell culture, HaCaT cells were exposed to UVB radiation (2.2 mJ/cm^2^), followed by treatment of 20 µL of HPs at 0.05 and 0.20 mg/mL for 24 h, respectively. VC (200 µM) was used as a positive control. After that, cell viability was calculated according to the method in Section 2.5.1.

#### 2.5.3. Measurement of Intracellular ROS, MDA, and Antioxidant Enzymes

Following the protocol outlined by Cui et al. [29], HaCaT cells were subjected to UVB radiation at 2.2 mJ/cm^2^. Subsequently, cells were incubated with 20 µL of HPs at 0.05 and 0.20 mg/mL for 24 h. The cells underwent further treatment with aqueous HPs for 1 h in fresh medium. Following this, the cells were washed with PBS and exposed to 10 µM DCFH2-DA in fresh medium for 1 h. Qualitative images were captured using a TI-S inverted fluorescence biomicroscope (Nikon Co., Ltd., Tokyo, Japan), while quantitative analysis was conducted using ImageJ software(https://fiji.sc/, accessed on 8 September 2023).

SOD, CAT, and MDA levels were determined using the kits according to the manufacturer’s instructions [30].

#### 2.5.4. Measurement of Levels of Inflammatory Factors

TNF-α, IL-6, and IL-1β levels in UVB-irradiated cell were measured using the kit according to the manufacturer’s instructions.

#### 2.5.5. Measurement of Protein Expression

Western blot, following the method described by Chaiprasongsuk et al. [31], was employed to assess the expression of Nrf2, Keap1, NF-κB (P65), Phospho-NF-κB (P65), IκBα, Phospho-IκBα, and Toll-like receptor 4 (TLR4) proteins in HaCaT cells. Intranuclear and extranuclear proteins were extracted using RIPA buffer. After SDS-PAGE separation, the proteins were transferred to PVDF membranes, followed by closure with 1X rapid closure solution (Shanghai Yase Biomedical Technology Co., Ltd., Shanghai, China) for 15 min. Subsequently, PVDF membranes were incubated for 12 h at 4 °C with primary antibodies, and horseradish peroxidase-conjugated secondary antibodies were applied for 60 min at 37 °C. The PVDF membranes were then subjected to a 1-h incubation at 4 °C using the ultrasensitive ECL chemiluminescence reagent (Biyun Tian Biotechnology Co., Ltd., Shanghai, China). Following development, the protein expression density was quantified and normalized to GADPH, expressed as a ratio.

### 2.6. Molecular Docking Experiments of Nrf2 with NF-κB (P65)

The IDs of the PBDs of Nrf2 and NF-κB (P65) were found through the RCSB PDB protein structure website (https://www.rcsb.org/, accessed on 18 October 2023), and the IDs of their PBDs were (Nrf2: 2FLU, NF-κB (P65): 4Q3J) [19,32]. In addition, the 3D compound structures, including chlorogenic acid (CID: 1794427), quercetin (CID: 5280343), isorhamnetin (CID: 5281654), and luteolin (CID: 5280445), were obtained from PubChem Download (https://pubchem.ncbi.nlm.nih.gov/, accessed on 18 October 2023). Affinities were obtained by docking analysis with AutoDock Vina and Pymol under default settings [33]. The docking results were visualized using Discovery Studio to obtain 2D vs. 3D plots.

### 2.7. Statistical Analysis

All the data were expressed as the mean ± SD (n = 3) and analyzed by an ANOVA test using SPSS 27.0.1. Duncan’s multiple range test was used to analyze the significant differences between the means of parameters (*p* < 0.05, *p* < 0.01, or *p* < 0.001).

## 3. Results

### 3.1. Chemical Analysis of HPs by LC–MS/MS

#### Total Ion Chromatogram of HPs

As shown in Figure S1, we conducted a comprehensive analysis of the phytochemical composition of HPs using LC–MS/MS in both positive and negative ion modes. Following the analysis, the acquired data underwent processing through the ProgenesisQI metabolomics software (https://www.nonlinear.com/progenesis/qi/, accessed on 8 September 2023). Compound identification was meticulously carried out by consulting databases, specifically (http://www.hmdb.ca/, accessed on 8 September 2023) and (https://metlin.scripps.edu/, accessed on 8 September 2023). The outcomes of this analysis, detailing the chemical composition, are meticulously documented in Table 1. Fifteen main components identified from HPs were mainly dominated by flavonoids and phenylpropanoid compounds, and studies have shown that flavonoids and phenylpropanoid compounds showed remarkable antioxidative and anti-inflammatory functions [28,34], especially the following four compounds.

**Figure 1:**
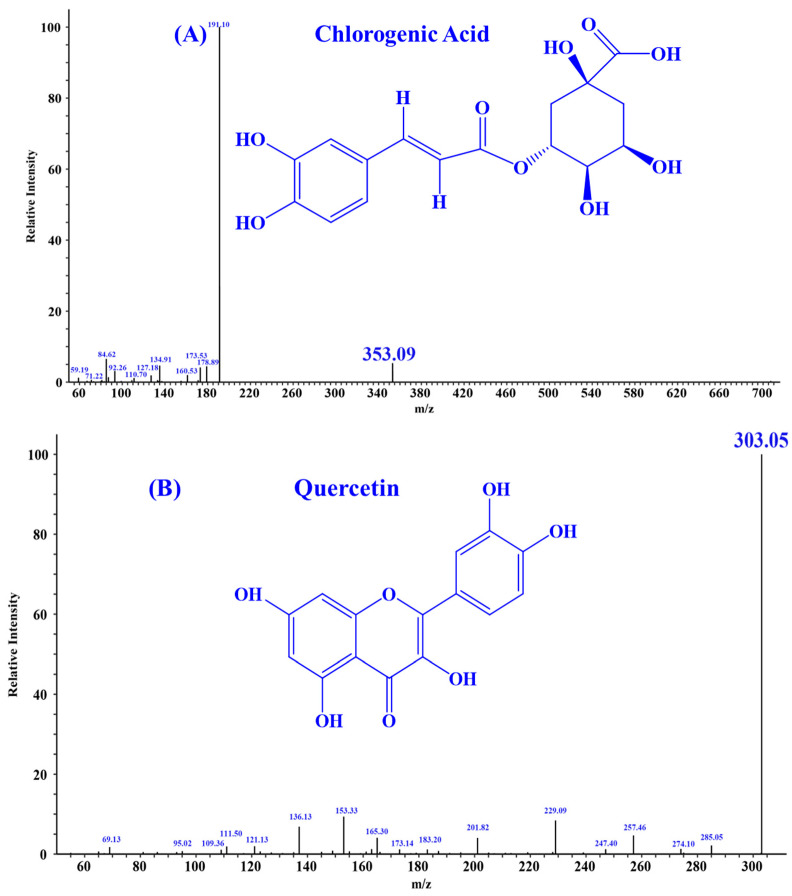

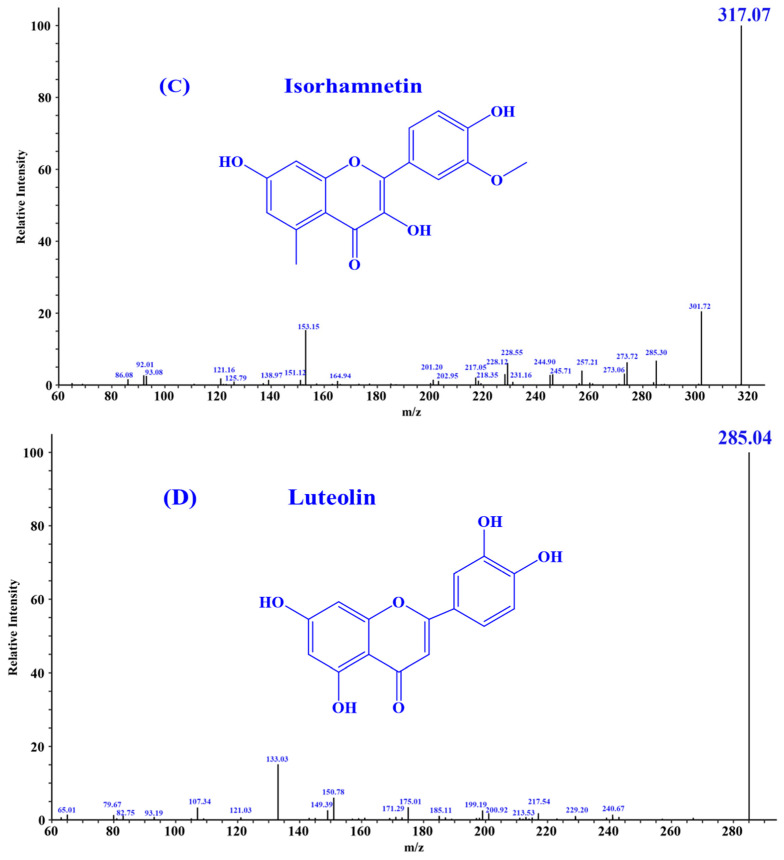
Secondary mass spectra of four important substances identified from HPs. (**A**) Chlorogenic Acid; (**B**) Quercetin; (**C**) Isorhamnetin; (**D**) Luteolin.

According to the literature, quercetin [18], isorhamnetin [19], luteolin [35], and chlorogenic acid [36] may be four key substances in HPs, and should have important contributions to the activity of HPs, especially antioxidative and anti-inflammatory functions. In addition, the secondary mass spectra of quercetin (*m*/*z* 303.05, Formula C_15_H_10_O_7_), isorhamnetin (*m*/*z* 317.07, Formula C_16_H_12_O_7_), luteolin (*m*/*z* 285.04, Formula C_15_H_10_O_6_), and chlorogenic acid (*m*/*z* 353.09, Formula C_16_H_18_O_9_) were depicted in Figure 1.

### 3.2. Effect of HPs on DPPH Radical Scavenging Activity

**Figure 2:**
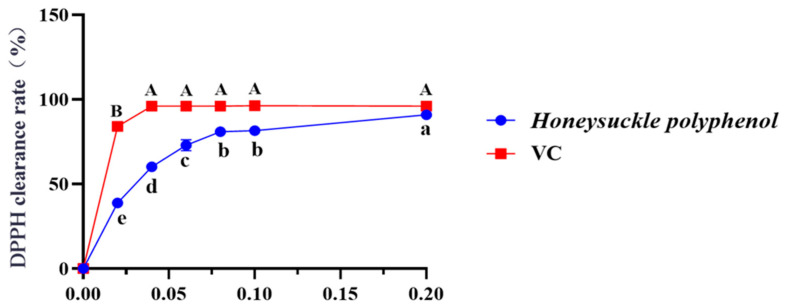
Effects of HPs (0.04–0.20 mg/mL) on DPPH radical scavenging rate. Ascorbic acid (VC) was used as a positive control. A, B or a–e Same letters indicated that the differences were not statistically significant (*p* > 0.05).

Figure 2 depicted that the DPPH radical scavenging rate of HPs at 0.04 mg/mL reached 60.16 ± 0.30%. Additionally, the DPPH scavenging rate of HPs at 0.04–0.20 mg/mL showed a steady increase in a dose-dependent manner. Its scavenging rate (90.94 ± 0.08%) at 0.20 mg/mL was comparable to that of the positive control. The present results indicated that HPs had a significant DPPH radical scavenging effect, reflecting its better antioxidant activity.

### 3.3. Mitigating Function of HPs on UVB-Irradiated HaCaT Cells through Antioxidant Activity

#### 3.3.1. Establishment of UVB-Irradiated Cell Model

**Figure 3:**
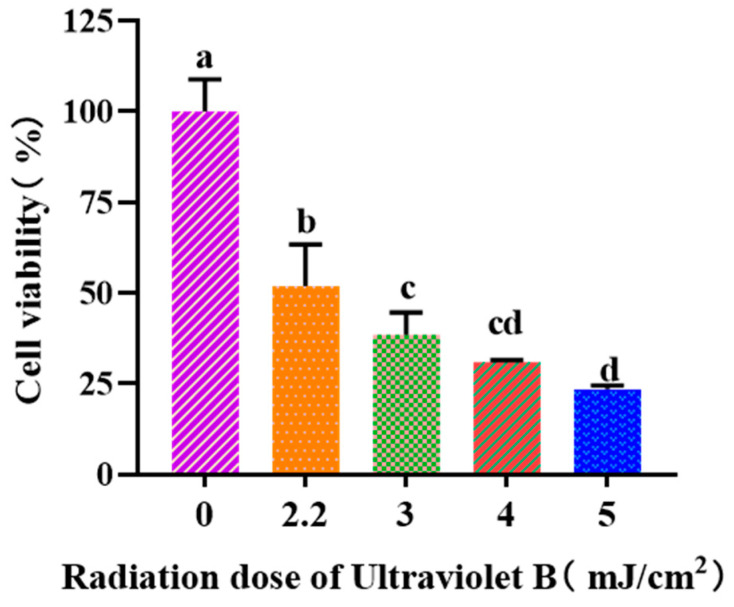
Effects of UVB radiation doses on the viability of HaCaT cells. a–d Same letters suggested that the differences were not statistically significant (*p* > 0.05).

Figure 3 depicted that the viability of HaCaT cells was gradually weakened when the UVB dose was increased from 0 to 5.0 mJ/cm^2^. When the UVB radiation dose was 2.2 mJ/cm^2^, the cell viability was 51.97 ± 11.46%. According to the report by Hu et al. [37], the optimal radiation dose to establish the UVB-irradiated cell model was determined based on half of the lethal radiation intensity. Consequently, a dosage of 2.2 mJ/cm^2^ was selected to establish the UVB-irradiation model of HaCaT cells.

#### 3.3.2. Effects of HPs on the Viability of UVB-Irradiated HaCaT Cells

**Figure 4:**
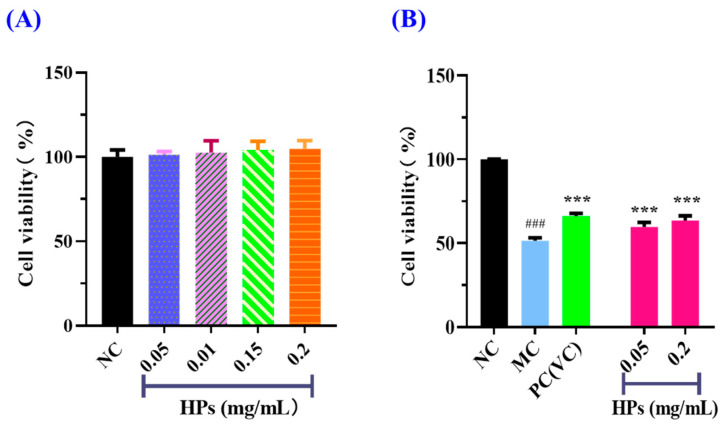
Effects of HPs on the viability of HaCaT cells (**A**) and UVB-irradiated HaCaT cells (**B**). ^###^ *p* < 0.001 vs. blank control group (NC); *** *p* < 0.001 vs. model group (MC).

According to the results in Figure 4A, the cell viability of the HP (0.05–0.20 mg/mL) groups did not present a significant difference with that of the blank group (*p* > 0.05), suggesting that HPs have no toxic effects on HaCaT cells. Therefore, 0.05 and 0.20 mg/mL were selected as the low and high doses of HPs in subsequent experiments. The results in Figure 4B depicted the same indication that HPs were not toxic to HaCaT cells under the tested conditions. The survival rates of HaCaT cells treated with HPs at 0.05 (low dose) and 0.20 mg/mL (high dose) were 59.71 ± 2.75% and 63.58 ± 2.69%, respectively, which were remarkably higher than that of the model group (51.41 ± 1.81%) (*p* < 0.001). These results clearly indicated that HPs could contribute to the repair of the UVB-irradiated cell damage.

#### 3.3.3. Effects of HPs on ROS Levels in UVB-Irradiated HaCaT Cells

**Figure 5:**
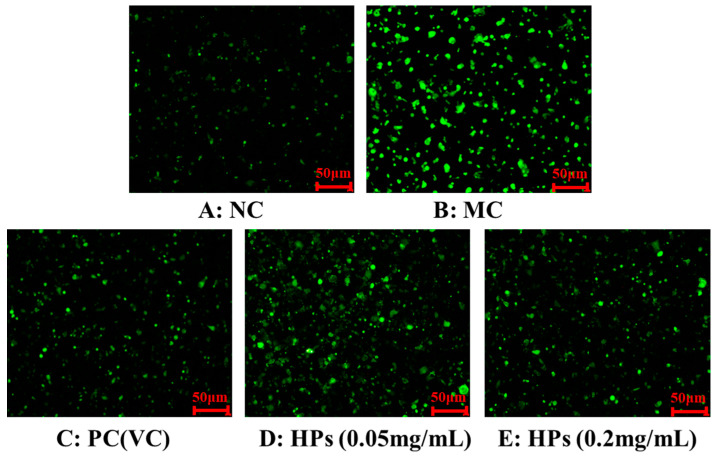
DCFH-DA staining method to determine intracellular ROS content. VC was used as a positive control. (**A**): NC (Normal Control); (**B**): Model (UVB-irradiated model of HaCaT cells); (**C**): PC(VC) (Positive control); (**D**): HPs (0.05 mg/mL) (Low dose group of HPs); (**E**): HPs (0.20 mg/mL) (High dose group of HPs).

**Figure 6:**
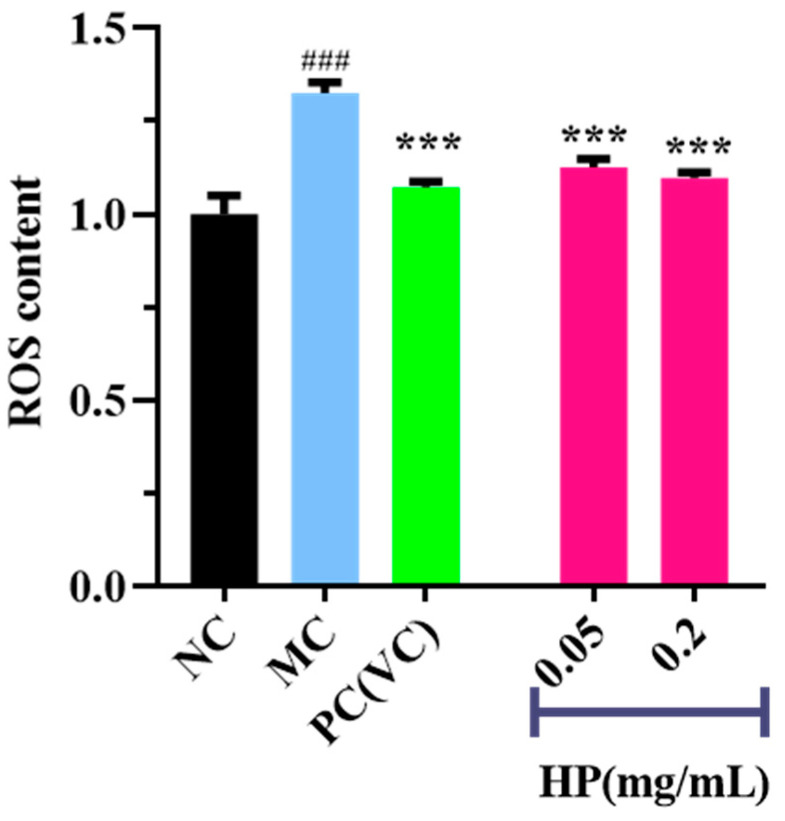
Effects of low (0.05 mg/mL) and high (0.20 mg/mL) doses of HPs on ROS levels in UVB-irradiated HaCaT cells. VC was used as positive control. ^###^ *p* < 0.001 vs. NC, *** *p* < 0.001 vs. MC.

Figure 5 and Figure 6 show the effects of HPs on ROS levels in the UVB-irradiated model of HaCaT cells. The fluorescence intensity and area of the model group (Figure 5B) increased compared with that of the blank group (Figure 5A), proving a remarkable increase in the intracellular ROS. Compared to the model group, the fluorescence area and intensity of the HP groups (Figure 5D,E) decreased with the increasing concentration of HPs, demonstrating a significant reduction in intracellular ROS. Figure 6 accurately quantifies the effects of HPs on ROS content in UVB-irradiated cells, and both low (0.05 mg/mL) and high (0.20 mg/mL) doses of HPs showed a highly reducing ability to decrease ROS levels (*p* < 0.001).

#### 3.3.4. Effects of HPs on Intracellular Oxidase and Oxide Levels in UVB-Irradiated HaCaT Cells

**Figure 7:**
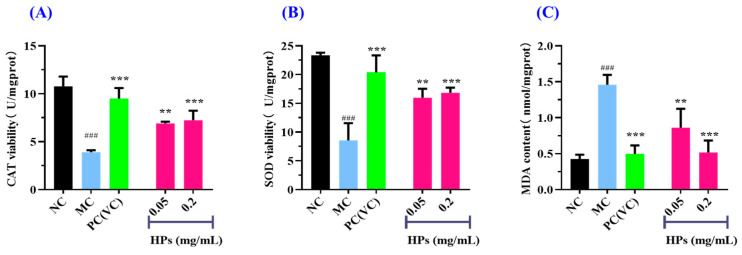
Effects of low (0.05 mg/mL) and high (0.20 mg/mL) doses of HPs on CAT (**A**), SOD (**B**), and MDA (**C**) levels in UVB-irradiated HaCaT cell model. VC was used as positive control. ^###^ *p* < 0.001 vs. NC; ** *p* < 0.01 and *** *p* < 0.001 vs. MC.

Figure 7A,B depict that the activities of antioxidant enzymes (SOD and CAT) in the HP groups increased gradually when the concentration of HPs increased from 0.05 mg/mL to 0.20 mg/mL. At 0.20 mg/mL, the CAT and SOD activities in the HP groups were 7.23 ± 0.99 and 16.83 ± 0.89 U/mg prot, respectively, which were markedly higher than those of the model group (*p* < 0.001). Figure 7C depicts that the content of MDA decreased gradually when the concentration of HPs increased from 0.05 mg/mL to 0.20 mg/mL. At 0.20 mg/mL, the MDA content decreased to 0.52 ± 0.17 nmol/mg prot, which was significantly higher than that of the model group (*p* < 0.001).

#### 3.3.5. Effect of HPs on the Antioxidant Proteins in UVB-Irradiated HaCaT Cells

As a pivotal transcription factor, Keap/Nrf2 regulates the cellular defense system against oxidative damage [31,38]. Under normal conditions, Keap1/Nrf2 resides outside the nucleus. However, when confronted with oxidative stress, Nrf2 dissociates from Keap1, facilitating its translocation into the nucleus. This is followed by the transcription and translation of antioxidant enzymes, which ultimately strengthen the cell’s defense against oxidative damage [39]. Therefore, the reparative function of HPs in UVB-irradiated HaCaT cells was assessed by investigating the expression levels of the Nrf2 protein inside and outside the nucleus.

**Figure 8:**
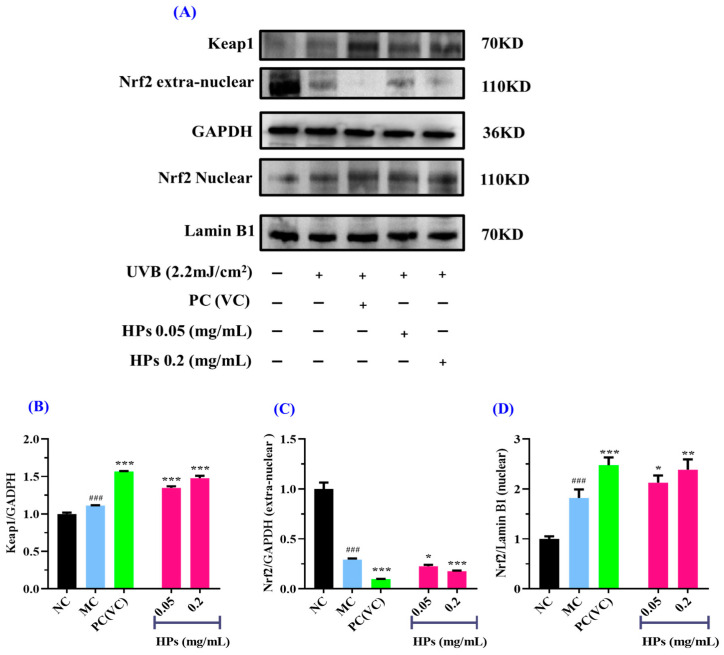
Effects of low (0.05 mg/mL) and high (0.20 mg/mL) doses of HPs on the expression of Keap1 and Nrf2 proteins in UVB-irradiated HaCaT cell model. VC was used as positive control. (**A**) Western-blot results of Keap1 and Nrf2; (**B**) Keap1 protein; (**C**) Nrf2 protein outside the nucleus; (**D**) Nrf2 protein inside the nucleus. ^###^ *p* < 0.001 vs. NC; * *p* < 0.05, ** *p* < 0.01, and *** *p* < 0.001 vs. MC.

In comparison to the normal group, the expression of the Keap1 protein was increased strikingly in the model group after being subjected to oxidative stress (*p* < 0.001) (Figure 8B). When the concentration of HPs was at 0.05 and 0.20 mg/mL, Keap1 expression was significantly increased in comparison to the model group (*p* < 0.001), indicating that Keap1 dissociates from Nrf2, thereby promoting antioxidant ability (Figure 8B).

Moreover, the expression of the Nrf2 protein outside the nucleus in the model group showed a remarkable decline in comparison with that in the normal group (*p* < 0.001) (Figure 8C), suggesting that UVB-irradiated damage to HaCaT cells prompted Nrf2 entry into the nucleus. When the HP concentration was at 0.20 mg/mL, there was a highly significant reduction in Nrf2 protein expression in comparison to the model group (*p* < 0.001) (Figure 8C). As shown in Figure 8D, for the expression of Nrf2 in the nucleus, there was a remarkable rise in the model group of UVB-irradiated HaCaT cells in comparison with the normal group (*p* < 0.001), and HPs could remarkably improve the expression of the Nrf2 protein in comparison with the model group (*p* < 0.05 or 0.01). In conclusion, HPs can promote the expression of the Nrf2 protein into the nucleus and thus reduce the UVB-induced cell damage.

#### 3.3.6. Molecular Docking of Chlorogenic Acid, Quercetin, Isorhamnetin, and Luteolin to Nrf2

Under normal conditions, the Nrf2 protein predominantly resides in the cytoplasm, forming a complex with the Keap1 protein. However, in the presence of an excess of ROS within the cells, these ROS interact with Cys residues on the Keap1 protein, leading to the modification and phosphorylation of Keap1. This phosphorylation event disrupts the association between Keap1 and Nrf2, ultimately leading to the liberation of Nrf2 from Keap1 [40]. Furthermore, antioxidant substances can participate in this process. They have the ability to compete for binding sites in Keap1 protein with Nrf2 protein, which further promotes Nrf2 separating from Keap1 and augments the pool of free Nrf2 protein entering the nucleus [41,42]. Upon nuclear translocation, Nrf2 can bind to antioxidant response elements (ARE), activating the transcription and translation of downstream antioxidant enzymes. This orchestrated cascade contributes significantly to the reinforcement of cellular antioxidant defense mechanisms [30,43].

In Figure 8, HP increases the content of Nrf2 protein in the nucleus. For elucidating the mechanism of HPs, a molecular docking experiment was used to predict the interactions of Nrf2 with four key components, including chlorogenic acid, quercetin, isorhamnetin, and luteolin.

**Figure 9:**
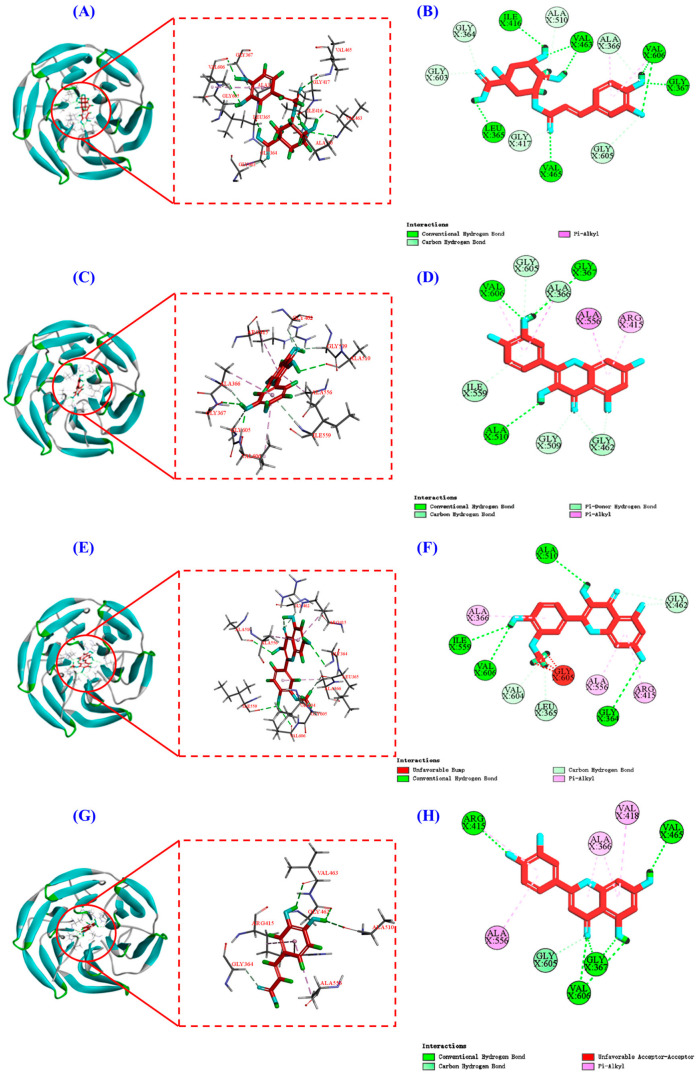
Molecular docking modeling of chlorogenic acid, quercetin, isorhamnetin, and luteolin with Keap1 protein. (**A**,**C**,**E**,**G**): 3D map of the interaction between Keap1 protein and chlorogenic acid, quercetin, isorhamnetin, and luteolin, respectively. (**B**,**D**,**F**,**H**): 2D plot of the interaction between Keap1 protein and chlorogenic acid, quercetin, isorhamnetin, and luteolin, respectively.

Keap1 (molecular weight 70 kDa) is a cysteine-rich protein comprising more than 625 amino acid residues, with a total of 27 Cys residues. The Kelch homologue (Kelch) structural domain of Keap1 interacts with the Neh2 structural domain of Nrf2 [44]. Thus, occupying the Kelch structural domain of Keap1, which competitively binds to Nrf2, can facilitate Nrf2 entry into the nucleus and initiate downstream antioxidant enzymes [45]. The two-dimensional diagram presented in Figure 9 elucidates the outcomes of molecular docking, demonstrating that four bioactive components of HPs, namely chlorogenic acid, quercetin, isorhamnetin, and luteolin, primarily engage through hydrogen bonding and electrostatic forces. Furthermore, as indicated in Table 2, the binding affinities of quercetin and isorhamnetin at specific sites (Arg415, Gly462, and Ala556) were determined to be −9.5 and −8.9 kcal/mol, while those of chlorogenic acid (Gly603) and luteolin (Arg415 and Ala556) were −8.6 and −9.4 kcal/mol, respectively. Consequently, the integrated insights derived from Figure 9 and Table 2 suggest that these four pivotal active compounds in HPs proficiently occupy the structural domain of Keap1’s Kelch homologue, competitively bind to Nrf2, facilitate Nrf2 binding, and ultimately enhance the translocation of Nrf2 to the nucleus. This comprehensive understanding provides robust molecular support for the regulatory role of HPs in the Nrf2 signaling pathway, offering novel avenues for the design and development of related bioactive components.

### 3.4. Effects of HPs on Intracellular Inflammatory Factors in UVB-Irradiated HaCaT Cells

#### 3.4.1. Effects of HPs on Intracellular TNF-α, IL-6, and IL-1β in UVB-Irradiated HaCaT Cells

**Figure 10:**
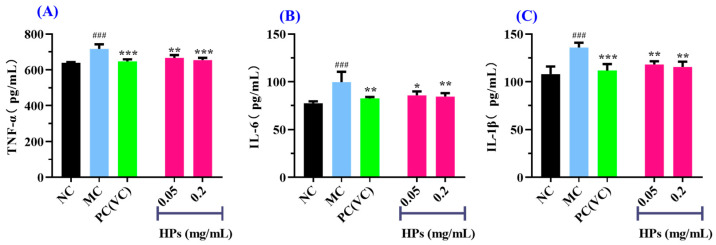
Effects of low (0.05 mg/mL) and high (0.20 mg/mL) doses of HPs on the levels of inflammatory factors TNF-α (**A**), IL-6 (**B**), and IL-1β (**C**) in UVB-irradiated HaCaT cells. ^###^ *p* < 0.001 vs. NC; * *p* < 0.01, ** *p* < 0.01, and *** *p* < 0.001 vs. MC.

It is widely acknowledged that TNF-α, IL-6, and IL-1β are transcriptionally regulated by the nuclear factor NF-κB [46]. These cytokines are commonly employed as biomarkers to assess inflammatory responses in cellular systems. As shown in Figure 10A–C, when the concentration of HPs was increased from 0.05 mg/mL to 0.20 mg/mL, the levels of inflammatory factors were gradually decreased in UVB-irradiated HaCaT cells incubated with HPs. At 0.20 mg/mL, the level of TNF-α in the HP group was 654.36 ± 12.61 pg/mL, which was markedly lower than that of the model group (716.41 ± 26.09 pg/mL) (*p* < 0.001); Similarly, the levels of IL-6 and IL-1β were 84.57 ± 3.59 and 115.48 ± 5.65 pg/mL, markedly inferior to those of the model group (99.70 ± 10.75 and 135.91 ± 5.03 pg/mL, respectively) (*p* < 0.01).

#### 3.4.2. Effect of HPs on Inflammatory Protein Expression in UVB-Irradiated HaCaT Cells

NF-κB plays a key role in the regulation of inflammatory protein expression related to the inflammatory response [47]. TLR4 serves as an agonist for various inflammatory pathways, with the NF-κB pathway being one of them [48]. Activation of TLR4 results in the dissociation of NF-κB from IκBα, thereby promoting inflammation [49]. The predominant form of NF-κB is the p65:p50 heterodimer, and the activation of p65 is pivotal in the pathogenesis of numerous chronic diseases, including dermatitis-like conditions, inflammatory bowel disease, multiple sclerosis, and neurodegenerative disorders [50]. Consequently, the NF-κB p65 signaling pathway has become a focal point in drug discovery and development initiatives [51]. In unstimulated conditions, p65 binds to the inhibitory protein IκBα, remaining localized in the cytoplasm. Upon exposure to external stimuli, phosphorylation and dissociation of the inhibitory protein IκBα occur, allowing phosphorylated p65 to translocate into the nucleus. This process initiates downstream inflammatory factors, leading to the onset of inflammation [52].

**Figure 11:**
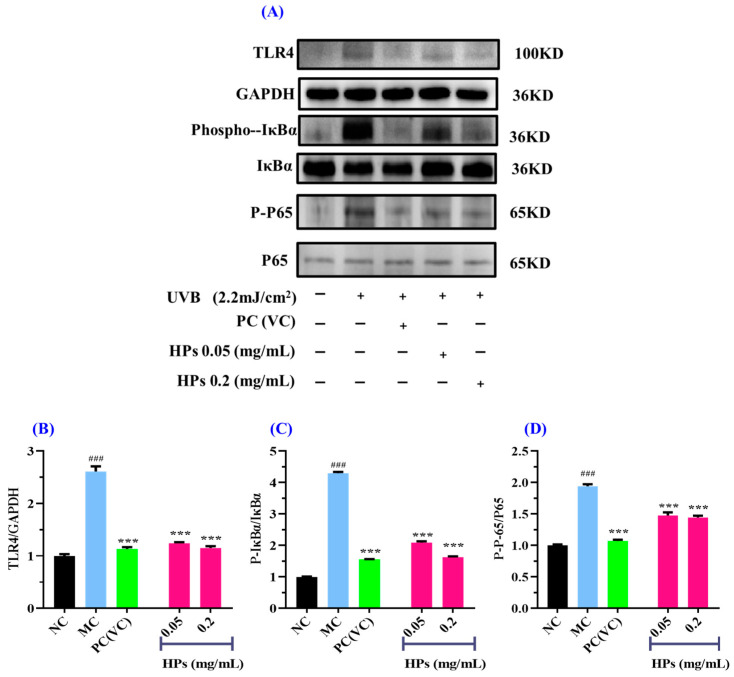
Effects of low (0.05 mg/mL) and high (0.20 mg/mL) doses of HPs on inflammatory NF-κB expression in UVB-irradiated HaCaT cells. VC was used as positive control. (**A**) Western-Blot; (**B**) TLR4 protein; (**C**) IκBα protein; (**D**) NF-κB (P65) protein. ^###^ *p* < 0.001 vs. NC; *** *p* < 0.001 vs. MC.

In Figure 11, we investigated the impact of HPs on NF-κB (P65) expression in UVB-irradiated HaCaT cells through both qualitative and quantitative protein blotting. In Figure 11B, TLR4 protein expression exhibited a remarkable growth (*p* < 0.001) in comparison to the normal group, indicating elevated TLR4 expression due to UVB damage. This heightened TLR4 expression subsequently led to the downstream activation of NF-κB (P65) and the production of inflammatory factors [53]. Following treatment with HPs at concentrations of 0.05 and 0.20 mg/mL, TLR4 protein expression showed a significant reduction (*p* < 0.001) compared to the model group, suggesting that HPs effectively inhibit TLR4 expression, thereby suppressing downstream inflammatory pathways. As depicted in Figure 11C,D, the protein expression of Phospho-IκBα (P-IκBα) and Phospho-NF-κB (P-P65) significantly increased in the model group compared to the normal group (*p* < 0.001). This indicates a substantial translocation of NF-κB (P65) into the nucleus, promoting the expression of inflammatory factors [54]. Upon treatment with HPs at 0.05 and 0.20 mg/mL, the expression of P-IκBα and P-P65 proteins demonstrated a significant reduction compared to the model group (*p* < 0.001). This signifies that HPs effectively inhibits the dissociation of IκBα from NF-κB (P65), thereby mitigating the production of inflammatory factors. In summary, our results indicated that HPs can effectively suppress inflammation in HaCaT cells induced by UVB irradiation through the NF-κB pathway.

#### 3.4.3. Molecular Docking of Chlorogenic Acid, Quercetin, Isorhamnetin, and Luteolin to NF-κB

Figure 11D indicated that HPs demonstrated a reduction in the expression level of the NF-κB (P65) protein within the nucleus. Then, the molecular docking method was employed to further elucidate the inhibition mechanism of HPs (chlorogenic acid, quercetin, isorhamnetin, and luteolin) on the NF-κB pathway.

**Figure 12:**
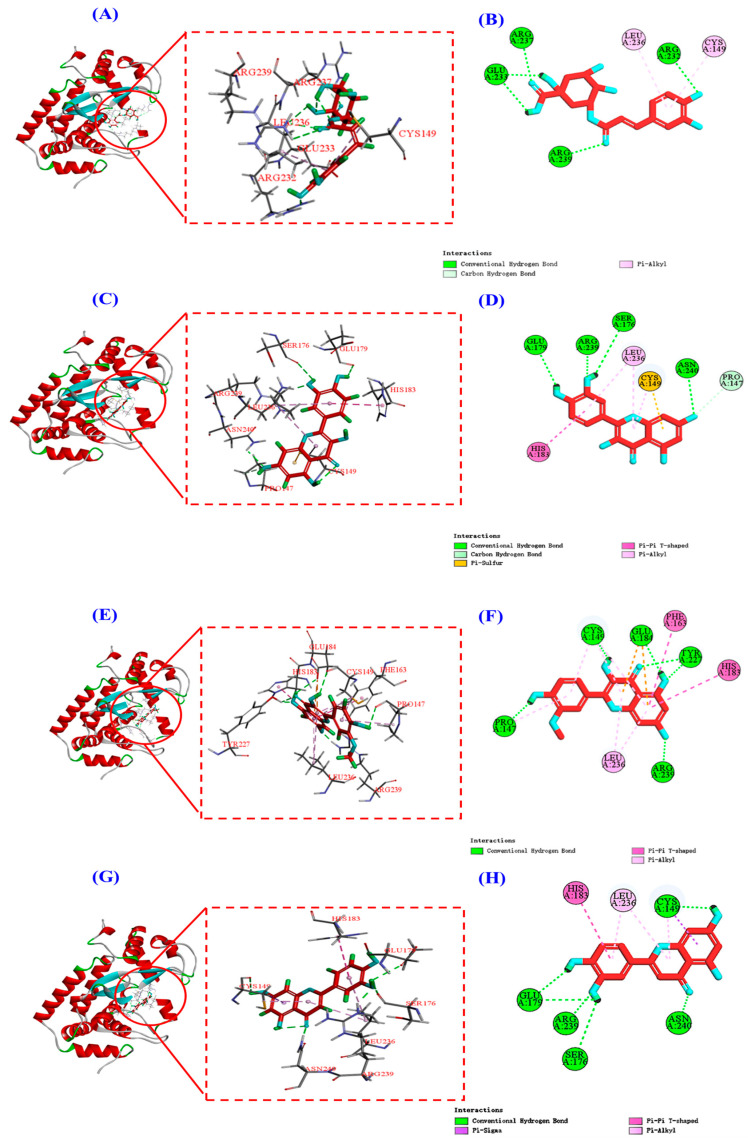
Molecular docking modeling of chlorogenic acid, quercetin, isorhamnetin, and luteolin with NF-κB (P65) proteins, respectively. (**A**,**C**,**E**,**G**): 3D map of NF-κB (P65) protein and chlorogenic acid (**A**), quercetin (**C**), isorhamnetin (**E**), and luteolin (**G**). (**B**,**D**,**F**,**H**): 2D plot of the interaction between NF-κB (P65) protein and chlorogenic acid (**B**), quercetin (**D**), isorhamnetin (**F**), and luteolin (**H**), respectively.

The 2D plots of the molecular docking results in Figure 12B,D,F,H show that chlorogenic acid, quercetin, isorhamnetin, and luteolin mainly interact with the NF-κB (P65) protein through hydrogen bonding, van der Waals’ force, and electrostatic force, and the specific forces and affinities are shown in Table 3. Combining the results of Figure 12 and Table 3, it can be seen that the four active substances of HPs inhibit the entry of NF-κB (P65) into the nucleus by occupying the amino acid residues on it [19]. It is also interesting to note that all four active substances have the same binding affinity of −7.7 kcal/mol to the NF-κB (P65) protein, which may be because the four active substances bind to the NF-κB (P65) protein mainly by hydrogen bonding and they all share a common hydrogen bonding interaction with Arg239.

## 4. Discussion

**Figure 13:**
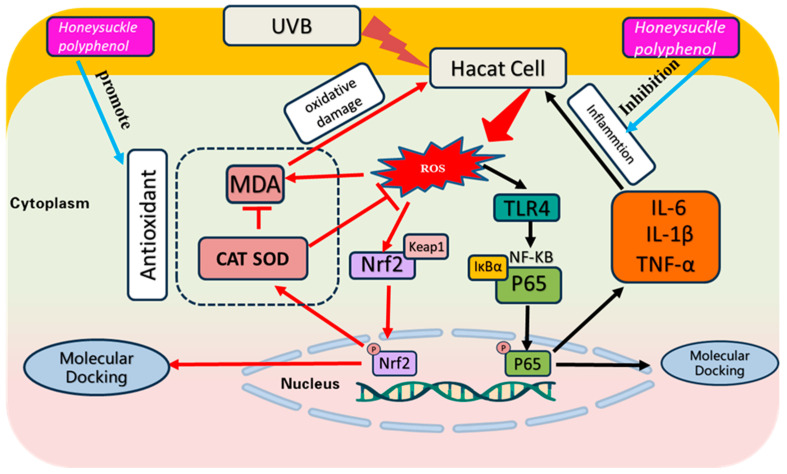
Effect of HPs on oxidative stress and inflammation production in UVB-irradiated model of HaCaT cells.

Exposing the skin to excessive UV radiation produces superfluous ROS, which further leads to oxidative damage to nucleic acids, lipids, and proteins, and triggers inflammation, resulting in redness and peeling of the skin [55,56]. In addition, acute UVB exposure leads to inflammation and oxidative damage in the stratum corneum, which in turn leads to epidermal damage and loss of barrier function [57]. Therefore, inhibiting UVB-induced skin damage provides a rational basis for treating UVB-induced diseases [58]. In this research, we discussed the reparative ability of HPs on UVB-damaged cells in terms of both antioxidant and inflammation inhibition (Figure 13).

Under UVB induction, HaCaT cells exhibit an overproduction of ROS, disrupting the redox equilibrium and diminishing the activity of antioxidative enzymes [59]. The accumulation of ROS and oxidized metabolites results in intracellular lipid peroxidation, wherein MDA, an end product of lipid peroxidation, compromises the structure and function integrality of cell membranes [60]. Consequently, antioxidant therapy has become a promising approach to counteract oxidative stress and enhance skin cell functionality by mitigating ROS-induced damage. In recent years, diverse compounds, including herbal medicines and plant extracts, have been explored for their potential in treating and preventing UV-induced skin damage, yielding some promising outcomes [61]. For the first time, this research assesses the cytoprotective effects of HPs against oxidative damage in UVB-irradiated HaCaT cells. The findings demonstrate a dose-dependent rise in SOD and CAT activities and a decline in MDA levels in UVB-reduced cells treated with HPs. These results imply the remarkable cellular repair potential of HPs, suggesting their capacity to ameliorate damage induced by UVB irradiation.

Relevant studies have elucidated that heightened intracellular levels of ROS may detrimentally impact the Nrf2 signaling pathway, consequently impeding the expression of antioxidant enzymes and phase II detoxification enzymes, ultimately contributing to oxidative damage [62]. Results from the Western blot analysis indicated that HPs showed a striking ability to effectively reverse these adverse effects by activating the Keap1/Nrf2 pathway, promoting the dissociation of Keap1 from Nrf2 protein, and facilitating the entry of Nrf2 into the nucleus. In addition, molecular docking experiments showed that four important substances from HPs, including chlorogenic acid, quercetin, isorhamnetin, and luteolin, occupied the active site of Nrf2 in the Kelch structural domain of the Keap1 protein. In summary, our findings demonstrate that HPs effectively inhibit the Keap1–Nrf2 coupling by binding to the active sites of Nrf2 and Keap1, thereby facilitating Nrf2 translocation into the nucleus and activation of Keap1/Nrf2 pathway that mitigates oxidative stress. This process culminates in the upregulation of antioxidant enzymes, phase II detoxification enzymes, and antioxidant genes, which collectively contribute to the maintenance of redox homeostasis and cellular protection. These results underscore the significant potential of HPs in promoting cell repair and protection, and warrant further investigation into their application for managing oxidative stress and maintaining skin health.

UV irradiation triggers an excessive accumulation of intracellular ROS, which not only causes oxidative damage to human skin but further triggers the development of an inflammatory response [63]. This link between oxidative stress and inflammation is of paramount importance in skin biology, with implications for skin health and disease. Our study demonstrated that HPs effectively modulated the production of IL-1β, IL-6, and TNF-α, resulting in a significant reduction in UV-induced skin inflammation. These results highlight the latent application of HPs as a therapeutic agent for skin disorders associated with UV-induced inflammation.

NF-κB is a versatile and redox state-dependent nuclear transcription factor that regulates the expression of a diverse set of genes involved in a variety of biological processes, including inflammatory factors [47]. ROS have been shown to cause an inflammatory response by stimulating the TLR4 receptor and activating the NF-κB pathway [2,64]. According to the results of the immunoblotting assay, HPs showed a better ability to inhibit the inflammatory response, which could be further inhibited by inhibiting the TLR4 receptor, thus dissociating NF-κB (P65) from IκBα and making the inflammatory response effectively controlled. In addition, from molecular docking experiments, it was shown that four important substances from HPs, including chlorogenic acid, quercetin, isorhamnetin, and luteolin, bind to NF-κB (P65) through electrostatic force, van der Waals force, and hydrogen bonding, and inhibit its entry into the nucleus, which suppresses inflammation [19,65,66]. As a result, we conclude that HPs reduce the synthesis of intracellular inflammatory factors by occupying the active site of NF-κB (P65) and preventing its entry into the nucleus, thereby decreasing inflammation-induced cellular damage. This discovery not only emphasizes the potential function of HPs in skin health but also lends significant support to HPs as a promising medication for skin inflammation healing. This gives an intriguing direction for future in-depth research.

## 5. Conclusions

In conclusion, we comprehensively elucidated the chemical composition of HPs and assessed their reparative effects on UVB-irradiated HaCaT cells. The cellular repair mechanisms orchestrated by HPs are primarily involved in activating the Keap1/Nrf2 pathway, resulting in an augmented cellular antioxidant capacity. Additionally, these polyphenols exhibited the capacity to suppress cellular inflammatory factors by down-regulating TLR4 and NF-κB (P65). Our findings establish a solid theoretical foundation for the efficacy of HPs in alleviating UVB irradiation-induced damage and offer valuable insights for the development of efficacious drugs to manage UV damage. Furthermore, rigorous scientific investigations are warranted to validate the practical applicability of HPs in animals as functional constituents in nutraceuticals, health foods, and cosmetics.

## Figures and Tables

**Table 1:** Chemical analysis of honeysuckle polyphenols (HPs) by LC–MS/MS.

Metabolite	Mode	*m*/*z*	Retention Time (min)	Formula	Fragmentation Score	CAS ID
3-O-Feruloylquinic acid	POS	369.12	3.31	C_17_H_20_O_9_	93.8	62929-69-5
Chlorogenic acid	NEG	353.09	3.76	C_16_H_18_O_9_	70.8	327-97-9
Quercetin	POS	303.05	4.04	C_15_H_10_O_7_	88.9	117-39-5
Isoquercitrin	POS	465.10	4.04	C_21_H_20_O_12_	97.2	482-35-9
Isorhamnetin	POS	317.07	4.45	C_16_H_12_O_7_	88.5	480-19-3
Kaempferol-3-O-rutinoside	POS	595.17	4.47	C_27_H_30_O_15_	95.5	17297-56-2
Kaempferol-3-O-glucoside	POS	449.11	4.48	C_21_H_20_O_11_	92.7	480-10-4
Hyperoside	POS	465.10	4.59	C_21_H_20_O_12_	97.7	482-36-0
Kaempferol	POS	287.06	4.61	C_15_H_10_O_6_	90.6	520-18-3
Quercetin-3-glucoside	POS	463.09	4.65	C_21_H_20_O_12_	94.9	482-35-9
Astragalin	POS	449.11	5.18	C_21_H_20_O_11_	91.5	480-10-4
Ferulic Acid	NEG	193.05	5.49	C_10_H_10_O_4_	98.5	537-98-4
Luteolin	NEG	285.04	5.56	C_15_H_10_O_6_	75.8	491-70-3
Genistein	POS	271.06	5.58	C_15_H_10_O_5_	90.7	529-59-9
Biochanin A	NEG	283.06	5.68	C_16_H_12_O_5_	94.3	491-80-5

**Table 2:** Molecular docking studies of chlorogenic acid, quercetin, isorhamnetin, and luteolin in complex with Keap1 protein and their binding energy.

Binding Ligand	Amino Acid Residue That Interacts	Docking Score
Chlorogenic Acid	Hydrogen bonding: Gly603	−8.6 kcal/mol
Quercetin	Hydrogen bonding: Gly462 Electrostatic interaction: Arg415 and Ala556	−9.5 kcal/mol
Isorhamnetin	Hydrogen bonding: Gly462 Electrostatic interaction: Arg415, and Ala556	−8.9 kcal/mol
Luteolin	Electrostatic interaction: Arg415, and Ala556	−9.4 kcal/mol

**Table 3:** Molecular docking studies of chlorogenic acid, quercetin, isorhamnetin, and luteolin in complex with NF-κB (P65) and their binding energy.

Binding Ligand	Amino Acid Residue That Interacts	Docking Score
Chlorogenic Acid	Hydrogen Bonding: Arg237, Arg232, Glu233, and Arg239 Electrostatic interaction: Leu263 and Cys149	−7.7 kcal/mol
Quercetin	Hydrogen bonding: Asn240, Glu179, Arg239, Ser176, and PRO147 Electrostatic interaction: Leu263 and His183 Hydrophobic force: Cys149	−7.7 kcal/mol
Isorhamnetin	Hydrogen bonding: Cys149, Glu184l Arg239, Tyr227, and PRO147 Electrostatic interaction: Leu263, His183, and Phe163	−7.7 kcal/mol
Luteolin	hydrogen bonding: Cys149, Glu179, Arg239, Ser176, and Asn240 Hydrophobic force: Leu263 and His183	−7.7 kcal/mol

## Data Availability

The original manuscript of this study is included in the article and further information is available upon reasonable request to the corresponding author.

## Supplementary Materials

The following supporting information can be downloaded at: https://www.mdpi.com/article/10.3390/antiox13030294/s1, Figure S1. Total ion chromatogram of honeysuckle polyphenols (HPs) by LC-MS/MS in positive and negative ion modes, with the horizontal coordinate indicating the retention time of the peaks and the vertical coordinate indicating the relative total ion intensity.

## Abbreviations

CAT, catalase; CCK8, Cell Counting Kit-8; DPPH, 2,2-diphenyl-1-picrylhydrazyl; HaCaT cells, human immortal keratinocyte cell line; HP, Honeysuckle polyphenol; IκBα, Inhibitor of Nuclear Factor Kappa-B Alpha; IL-1β, Interleukin-1 beta; IL-6, Interleukin-6; Keap1, Kelch-like ECH-associated protein 1; LC–MS/MS, liquid chromatography-tandem mass spectrometry; MDA, malondialdehyde; NF-κB, nuclear factor kappa-light chain enhancer of B cells; Nrf2, nuclear factor erythroid-2-related factor 2; OD, Optical Density; PBS, Phosphate-Buffered Saline; Phospho-IκBα, phosphorylated Inhibitor of Nuclear Factor Kappa-B Alpha; PVDF, polyvinylidene difluoride; ROS, reactive oxygen species; SDS-PAGE, Sodium Dodecyl Sulfate Polyacrylamide Gel Electrophoresis; SOD, superoxide dismutase; TLR4, Toll-like Receptor 4; TNF-α, Tumor Necrosis Factor-alpha; UVB, ultraviolet B; VC, Vitamin C.
