# Supplementary material for: Chemical Characterization of Honeysuckle Polyphenols and Their Alleviating Function on Ultraviolet B-Damaged HaCaT Cells by Modulating the Nrf2/NF-κB Signaling Pathways
Source: Antioxidants (Basel). 2024 Feb 28;13(3):294. doi: 10.3390/antiox13030294 (PMC10967407; doi:10.3390/antiox13030294)
Supplement: Supplementary file 1 [file antioxidants-13-00294-s001.zip › antioxidants-2864429-supplementary.pdf]

Supplementary Material

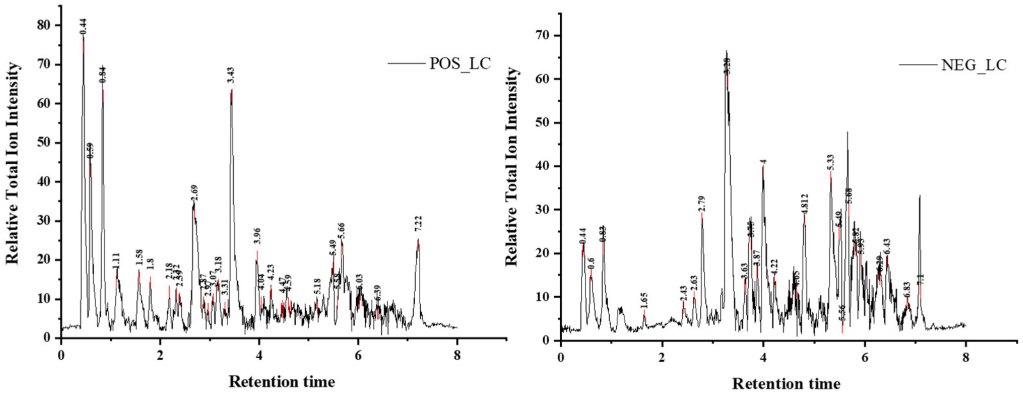

Figure S1. Total ion chromatogram of honeysuckle polyphenols (HPs) by LC-MS/MS in positive and negative ion modes, with the horizontal coordinate indicating the retention time of the peaks and the vertical coordinate indicating the relative total ion intensity.
